# Establishment of a Novel Miniature Double-Lumen Catheter Single-Cannulation Venovenous Extracorporeal Membrane Oxygenation Model in the Rat

**DOI:** 10.3390/membranes14030055

**Published:** 2024-02-20

**Authors:** Yutaka Fujii, Takuya Abe

**Affiliations:** Department of Clinical Engineering and Medical Technology, Niigata University of Health and Welfare, Niigata 950-3198, Japan; takuya-abe@nuhw.ac.jp

**Keywords:** extracorporeal circulation, venovenous extracorporeal membrane oxygenation (VV ECMO), double-lumen catheter, rat model

## Abstract

In recent years, venovenous extracorporeal membrane oxygenation (VV ECMO) has been used to support patients with severe lung disease. Active use of VV ECMO was also recommended for severe respiratory failure due to COVID-19. However, VV ECMO is also known to cause various complications due to extracorporeal circulation. Although we conducted ECMO research using rats, we have not been able to establish whether double-lumen single-cannulation VV ECMO models in rats have been described previously. The purpose of this study was to establish a simple, stable, and maintainable miniature double-lumen single-canulation VV ECMO model in rats. A double-lumen catheter used as a plain central venous catheter (SMAC plus Seldinger type; Covidien Japan Co., Tokyo, Japan) was passed through the right external jugular vein and advanced into the right atrium as a conduit for venous uptake. The VV ECMO system comprised a roller pump, miniature membrane oxygenator, and polyvinyl chloride tubing line. During VV ECMO, blood pressure and hemodilution rate were maintained at around 80 mmHg and 30%, respectively. Hemoglobin was kept at >9 g/dL, no serious hemolysis was observed, and VV ECMO was maintained without blood transfusion. Oxygenation and removal of carbon dioxide from the blood were confirmed and pH was adequately maintained. This miniature VV ECMO model appears very useful for studying the mechanisms of biological reactions during VV ECMO.

## 1. Introduction

Extracorporeal life support, such as extracorporeal membrane oxygenation (ECMO), is an essential technology for treating circulatory and respiratory failure [1,2]. In recent years, venovenous ECMO (VV ECMO) has increasingly been used to support patients with severe lung disease [3,4,5]. Active use of VV ECMO was also recommended for severe respiratory failure resulting from COVID-19 [6,7]. On the other hand, VV ECMO is known to cause various complications because it is highly invasive [8,9]. Although there are many clinical studies on VV ECMO, basic research remains relatively sparse. In addition, continued large-scale animal experiments are difficult due to economic and ethical issues. Given this background, a miniature VV ECMO model is desirable to facilitate studies into the mechanisms of biological reactions in the circulation during VV ECMO. We have already established a rat model of VV ECMO [10]. However, that model shows a relatively high degree of surgical invasiveness, using two single-lumen cannulas for the dual-cannulation ECMO model [10], and thus does not represent a model of cannulation with a single dual-lumen cannula for VV ECMO, as is being actively introduced worldwide for the treatment of severe respiratory diseases in humans. In addition, an experimental mouse and large-scale animal model have been reported [11,12], but no double-lumen single-cannulation rat VV ECMO models in rats have been described. The purpose of this study is to establish a rat VV ECMO model with single cannulation of a double-lumen catheter in which blood gases, pH, and electrolytes can be stably maintained. We believe that establishing this miniature VV ECMO model will be very useful for studying the mechanisms underlying biological reactions during VV ECMO.

## 2. Material and Methods

### 2.1. Animals

This study was conducted with the approval of the Niigata University of Health and Welfare Animal Care and Use Committee (ethics approval code: 30009–02016 and 22003). All procedures were performed in accordance with the National Institutes of Health guidelines for animal care. Subjects were male Sprague–Dawley rats (bodyweight, 400–450 g; Japan SLC Inc., Shizuoka, Japan). Three rats were housed in each cage under a 12 h light–dark cycle with food and water available ad libitum.

### 2.2. Anesthesia, Surgical Preparation, and VV ECMO

**Figure 1:**
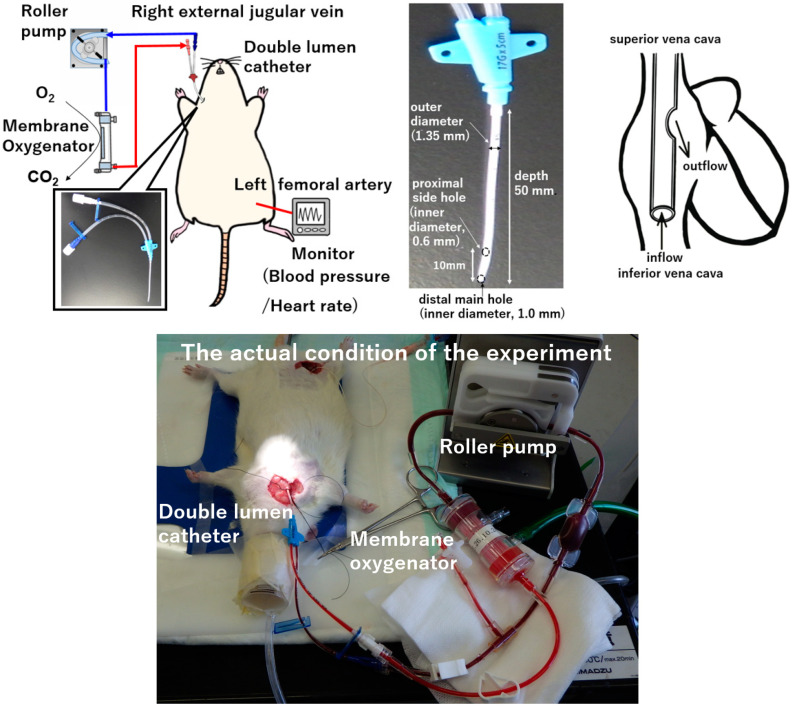
Double-lumen catheter, single-cannulation VV ECMO in a rat model.

After inducing anesthesia by inhalation of 5.0% isoflurane in oxygen-enriched air using a vaporizer, the rat was placed in a supine position and a rectal temperature probe was inserted. The animal was then further anesthetized with isoflurane mask narcosis and spontaneous breathing was maintained with an oxygen fraction of 21%. Isoflurane at 2.0–2.5% was used to maintain anesthesia (without neuromuscular blocking agents), and rectal temperature was maintained at 35–36 °C. The right femoral artery was cannulated with SP-31 polyethylene tubing (Natsume Seisakusho Co., Tokyo, Japan) for monitoring of arterial blood pressure using a Power-Lab system (Model ML870; AD Instruments Japan Inc., Nagoya, Japan). For cannulation, a lateral skin incision on the right side of the neck was made to expose the right external jugular vein. A 4-0 silk suture (braid silk; Hashimoto Co., Tokyo, Japan) was placed cranially to ligate the distal side, with a slip knot placed at the proximal side of the vein. A double-lumen catheter used as a plain central venous catheter (SMAC plus Seldinger type; Covidien Japan Co., Tokyo, Japan) was passed through the right external jugular vein and advanced into the right atrium as the conduit for venous uptake. The double-lumen catheter was then secured using slip knots. This polyurethane-based double-lumen catheter consists of a distal main hole (inner diameter, 1.0 mm) and proximal side holes (inner diameter, 0.6 mm) with an outer diameter of 1.35 mm and a depth of 50 mm. After cannulation, anticoagulation was induced with an intravenous heparin sodium bolus (500 IU/kg). The VV ECMO system comprised a roller pump (REGLO Digital MS-2/6; ISMATEC, Wertheim, Germany), a miniature membrane oxygenator (tube connection part: inner diameter, 2.0 mm, outer diameter of 3.3 mm, Senko Medical Instrument Mfg. Co., Tokyo, Japan), and a polyvinyl chloride tubing circuit (Senko Medical Instrument Mfg. Co.). The miniature membrane oxygenator used in this study was developed for use in small animals. The hollow fiber material of the miniature membrane oxygenator was polypropylene. The membrane surface area was 0.039 m^2^, and it was a system in which blood flowed inside the hollow fibers. The model is approximately one fiftieth the size of that used for children in actual clinical practice, and considering the weight of the rat, it can simulate actual clinical practice in terms of size balance. The VV ECMO system was primed with 7.5 mL of saline with 0.5 mL (500 IU) of heparin. Figure 1 shows the experimental conditions.

### 2.3. Experimental Design

A total of fourteen Sprague–Dawley rats were randomized and divided into a sham group (*n* = 7) and a VV ECMO group (*n* = 7). The sham group underwent identical surgical procedures to the VV ECMO group, but no VV ECMO was commenced. VV ECMO pump flow was initiated and maintained at 50–60 mL/kg/min, and oxygen was added into the oxygenator during VV ECMO at a concentration of 100% (pump flow:oxygen flow = 1:10). Blood samples were collected in 0.5 mL at three defined time points: before VV ECMO (pre-VV ECMO), 60 min after initiation of VV ECMO, and 120 min after initiation of VV ECMO (end-VV ECMO). Blood sample volume was replaced with saline. Arterial blood gases (femoral artery), pH, electrolyte (sodium, potassium, chloride ions), and hemoglobin (Hb) concentration were also measured (VetStat Electrolyte and Blood Gas Analyzer; IDEXX Laboratories, Westbrook, NJ, USA). To confirm the presence or absence of hemolysis, free Hb was measured at the end of VV ECMO using the HemoCue^®^ Plasma/Low Hb System (HemoCue AB, Ängelholm, Sweden). All animals were euthanized at the end of the experiment by potassium chloride injection into the myocardium.

All data are expressed as mean ± standard deviation. The Mann–Whitney U-test was used for subsequent comparisons between groups at the same time points. All statistical analyses were performed using Stat-View version 5.0 (Abacus Concepts, Berkeley, CA, USA). Values of *p* < 0.05 were considered significant.

## 3. Results

**Figure 2:**
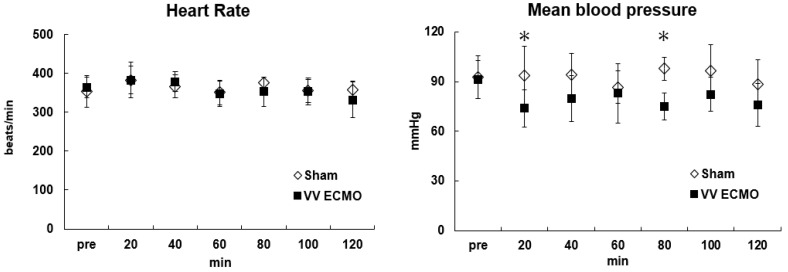
Hemodynamic variables (heart rate, mean arterial pressure) before and during VV ECMO. * *p* < 0.05 versus sham group at the same time point.

Table 1 shows changes in arterial blood gases, pH, electrolyte (sodium, potassium, chloride ions) and hemoglobin (Hb) concentration in each group during the experiment. Figure 2 shows changes in hemodynamic variables (heart rate, mean arterial pressure) before and during VV ECMO. During VV ECMO, blood pressure and hemodilution rate were maintained at around 80 mmHg and 30%, respectively. Hb was kept >9 g/dL and VV ECMO was maintained without blood transfusion. Plasma-free hemoglobin, an indicator of hemolysis, was below the detection limit value in all rats in the VV ECMO group, and no serious hemolysis was observed. Oxygenation and carbon dioxide removal from the blood were confirmed.

Specifically, PaO_2_ was stably maintained at approximately 200 mmHg and PaCO_2_ at approximately 35 mmHg, respectively, in the VV ECMO group throughout the experimental period. Similarly, pH was adequately maintained. Although the electrolyte concentrations were maintained within the normal range in both groups, a slight increase in potassium in the VV ECMO group was observed toward the end of the experiment. No problems were seen with this miniature VV ECMO system and extracorporeal circulation progressed safely. On the other hand, temporary poor blood removal was observed in several VV ECMO group rats. In this case, we were able to correct the problem by adjusting the position of the double-lumen catheter and continuing the experiment.

## 4. Discussion

Extracorporeal life support, such as in cardiopulmonary bypass and VA ECMO, is an essential tool for treating cardiorespiratory failure [1,2,13]. Recently, in isolated severe respiratory failure, VV ECMO has been the therapy of choice as a bridge to either recovery or transplantation [3,4,5]. Specifically, indications for VV ECMO include severe acute respiratory distress syndrome and exacerbation of chronic obstructive pulmonary disease [14,15]. In addition, active use of VV ECMO was recommended for severe pneumonia caused by N1H1 in 2009 and COVID-19 in 2020 [6,7]. Although clinical data are being collected, we believe that a highly versatile small animal VV ECMO model is necessary for the advancement of basic research.

We have already described an VV ECMO model for the rat [10]. In the present study, a novel miniature double-lumen single-cannulation rat model of VV ECMO was created by applying the previously established conventional rat extracorporeal circulation model. The VV ECMO model established in this study is a single cannulation model from the neck (right external jugular vein), which is the global standard and can be introduced with minimal surgical procedures, allowing for highly reproducible experiments. We confirmed that the hemodynamic parameters in rats were stable during VV ECMO. In contrast, in the VV ECMO group, there was a temporary decrease in blood pressure and a slight increase in potassium level at the end of the experiment. The decrease in blood pressure is thought to be due to an initial drop in blood pressure owing to the effects of blood dilution by the priming solution, and the increase in potassium cannot be ruled out as being caused by the destruction of blood cells by the roller pump. Based on the method for measuring free hemoglobin in this experiment (detection limit value of 30 mg/dL or less), it was judged that there was no severe hemolysis. However, in the case of disease, free hemoglobin of 5.9 mg/dL or more may be used as the standard. We believe that it is necessary to evaluate hemolysis in more detail. No double-lumen catheter single-cannulation VV ECMO methods have been described in small animal models worldwide. The construction of this rat VV ECMO model in this study was thus significant.

We suggest that the rat VV ECMO model is very useful for studying the mechanisms of biological reactions during VV ECMO and for basic studies of extracorporeal lung assistance devices. Further research is needed to elucidate the mechanisms of such biological reactions during VV ECMO. Specifically, we believe that research is necessary to effectively support the effects of temperature control during VV ECMO on the living body and changes in blood flow in major organs owing to increases and decreases in supplemental flow. VV ECMO is a special situation in which circulation maintenance depends on the body while supporting oxygenation. In particular, the right heart and pulmonary arteries are exposed to high oxygen partial pressure, to which they are not normally exposed, resulting in an unphysiological situation. We are developing a research plan focusing on the adverse effects of non-physiological hyperoxia management caused by VV ECMO. The detailed biological reactions during VV ECMO are unknown, and further research is required. Our established small animal model can be used in advanced research. In addition, long-term support reportedly did not confirm the effectiveness of ECMO when compared with conventional ventilator management and ECMO [16]. However, it should be noted that there was crossover between the control and ECMO groups. Furthermore, this trial had a small sample size. Patient conditions are not uniform, and evaluation of the effectiveness of ECMO requires further discussion. At the same time, we believe that basic research such as the evaluation of pathological conditions during ECMO is necessary. In our previous study, we used a rat to assess the gene expression of inflammatory cytokines in major organs during extracorporeal circulation [17]. In future research, differences in the implications of long-term VV ECMO and the effects of post-VV ECMO weaning need to be examined. In addition, we believe that by applying this model, it will be possible to construct an experimental system to verify rehabilitation effects during VV ECMO.

This study did show some limitations that merit consideration. We did not consider recirculation in the present VV ECMO model. The problem with recirculation is that oxygenated blood is sucked back into the ECMO circuit side before it circulates throughout the body, thereby reducing treatment efficiency. The possibility of recirculation cannot be excluded with this method of inserting a double-lumen catheter through the external jugular vein and placing it in the right atrium. In future research, we believe it is necessary to construct and examine a model that can measure the recirculation rate. This study led to basic research to develop a double-lumen catheter for VV ECMO that minimizes recirculation. However, catheter design and optimal placement position for the rodent model are some issues, and we are currently investigating ways to optimize the catheter structure using fluid simulation.

Another limitation was that this study used an internal perfusion membrane oxygenator. In this study, we believe that there are no major problems as effective gas exchange was achieved in the oxygenator and the pathological evaluation conducted in the pre-experiment revealed no material-related injuries, but this is an issue for future study. Currently, we are creating an externally perfused oxygenator in which blood circulates around the outside of the hollow fibers, simulating the extremely small membrane oxygenator used in clinical settings. We plan to conduct basic research to develop an artificial lung with an improved gas exchange efficiency. Since this experiment involved small animals, we used a roller pump that allows fine flow control, but centrifugal pumps are the mainstream tools used for VV ECMO in clinical situations. In addition, it was not possible to monitor the cardiac output in real time, and the support ratio was unknown. This is also a limitation of the present study.

In the future, we would like to study the living body and conduct basic research on VV ECMO systems and device development using this small animal model of VV ECMO. Specifically, we plan to develop effective double-lumen catheters for VV ECMO and coatings with strong antithrombotic and anti-inflammatory properties. In addition, we will also focus on membrane materials for hollow fibers in membrane oxygenators. High gas (carbon dioxide and oxygen) permeability and hydrophobicity are required for hollow fiber materials in membrane oxygenators. Currently, the mainstream materials for hollow fibers used in membrane oxygenators are polypropylene (porous membrane), silicone (homogeneous membrane), and polymethyl pentene (composite membrane), but we are also considering the application of other materials. The biocompatibility and antithrombotic properties of hollow fiber materials for membrane oxygenators are extremely important and require further study. We believe that the model developed in this study will also be useful for basic research on the development of circulation assistance devices, mainly artificial lungs. As no serious hemolysis was observed in this model, we believe that it is fully applicable to basic research. On the other hand, we believe that there are differences in biological reactions depending on the species, and we would like to consider this in the future.

## 5. Conclusions

In this study, a novel miniature double-lumen catheter single-cannulation VV ECMO model was established in the rat. This miniature VV ECMO model will be very useful not only for studying the mechanisms underlying biological reactions during VV ECMO but also for basic studies and the development of circulation assistance devices.

## Figures and Tables

**Table 1:** Arterial blood gas partial pressures, pH, electrolyte, and Hb concentrations before and during VV ECMO.

	Group	Pre	60 min	120 min
PaO_2_ (mmHg)	Sham	104 ± 9	101 ± 12	103 ± 11
	VV ECMO	106 ± 10	199 ± 35 *	186 ± 34 *
PaCO_2_ (mmHg)	Sham	40 ± 6	39 ± 6	38 ± 7
	VV ECMO	39 ± 5	35 ± 2 *	36 ± 3
pH	Sham	7.37 ± 0.07	7.35 ± 0.05	7.37 ± 0.09
	VV ECMO	7.38 ± 0.04	7.37 ± 0.07	7.38 ± 0.05
Na^+^ (mEq/L)	Sham	137 ± 2	138 ± 3	137± 2
	VV ECMO	137 ± 3	140 ± 4	139 ± 4
K^+^ (mEq/L)	Sham	4.2 ± 0.3	4.2 ± 0.4	4.3 ± 0.3
	VV ECMO	4.1 ± 0.2	4.8 ± 0.6	5.4 ± 0.4 *
Cl^−^ (mEq/L)	Sham	106 ± 3	107 ± 2	107 ± 3
	VV ECMO	107 ± 2	108 ± 4	109 ± 4
Hb (g/dL)	Sham	13.9 ± 1.2	13.3 ± 0.8	13.1 ± 1.4
	VV ECMO	13.8 ± 0.7	10.0 ± 0.9 *	9.2 ± 0.9 *

Variables are expressed by mean ± standard deviation. * *p* < 0.05 versus sham group at the same time. PaO_2_: partial pressure of arterial oxygen. PaCO_2_: partial pressure of arterial carbon dioxide. pH: power of hydrogen. Hb: hemoglobin.

## Data Availability

Data is contained within the article.

## Abbreviations

VV ECMO	Venovenous extracorporeal membrane oxygenation
VA ECMO	Venoarterial extracorporeal membrane oxygenation
PaO_2_	Arterial pressure of oxygen
PaCO_2_	Arterial pressure of carbon dioxide
Hb	Hemoglobin
